# National Utilization and Expenditure Trends of GLP-1 Receptor Agonists and Dual GLP-1/GIP Agonist in Croatia, 2017–2024

**DOI:** 10.3390/medicina61122210

**Published:** 2025-12-15

**Authors:** Mate Car, Damir Erceg, Mario Udovičić, Tomislav Bokun, Dario Rahelić

**Affiliations:** 1 Department of Primary Care & Public Health, Imperial College London, London SW7 2AZ, UK; 2 Centre for Data-Driven Policy and Management, Catholic University of Croatia, 10000 Zagreb, Croatia; 3 School of Medicine, Catholic University of Croatia, 10000 Zagreb, Croatia; 4 Department of Allergology and Immunology, “Srebrnjak” Children’s Hospital, 10000 Zagreb, Croatia; 5 Faculty of Medicine, Josip Juraj Strossmayer University of Osijek, 31000 Osijek, Croatia; 6 School of Medicine, University of Zagreb, 10000 Zagreb, Croatia; mario.udovicic@kbd.hr; 7 Department of Cardiology, University Hospital Dubrava, 10000 Zagreb, Croatia; 8 Department of Gastroenterology, Hepatology and Clinical Nutrition, University Hospital Dubrava, 10000 Zagreb, Croatia; tbokun@kbd.hr; 9 Faculty of Pharmacy and Biochemistry, University of Zagreb, 10000 Zagreb, Croatia; 10 Vuk Vrhovac University Clinic for Diabetes, Endocrinology and Metabolic Disorders, Merkur University Hospital, 10000 Zagreb, Croatia

**Keywords:** antihyperglycemic drugs, pharmacoepidemiology, GLP-1 receptor agonists, dual GLP-1/GIP agonist, SGLT-2 inhibitors, Croatia, drug utilization, pharmaceutical expenditure

## Abstract

*Background and Objectives*: GLP-1 receptor agonists (GLP-1 RAs; ATC A10BJ) and dual GLP-1/GIP agonist (ATC A10BX16) have expanded rapidly due to strong evidence in type 2 diabetes, obesity and metabolic dysfunction-associated steatotic liver disease (MASLD). Their high acquisition costs and accelerating uptake make them key drivers of pharmaceutical expenditure. This study quantified national utilization and expenditure trends for antihyperglycemic drugs in Croatia (2017–2024), with a focus on GLP-1 RA and dual GLP-1/GIP agonists. *Materials and Methods*: Aggregate national data on dispensed medicines, valued at wholesale pharmacy prices, were obtained from HALMED’s annual ATC/DDD reports. Utilization was expressed as defined daily doses per 1000 inhabitants per day (DDD/1000/day). We analyzed the total A10 and key subclasses. The dual GLP-1/GIP agonist was only marketed in 2024. Compound annual growth rates (CAGR) were calculated. *Results*: The total antihyperglycemic utilization increased from 66.9 to 96.8 DDD/1000/day (a 44.7% rise), while the total A10 expenditure increased from EUR 54.2 million to EUR 96.5 million, indicating that expenditure growth outpaced utilization growth. GLP-1 receptor agonist expenditure increased from EUR 5.2 million (2018) to EUR 28.6 million (2024) (CAGR 33.0%), reaching 29.8% of total A10 expenditure in 2024. Expenditure for GLP-1-based agents grew faster than their DDD volume because per-DDD acquisition costs are substantially higher than for SGLT-2 inhibitors. These growth patterns are consistent with trajectories reported in higher-uptake EU health systems, suggesting convergence rather than an outlier position for Croatia. *Conclusions*: Croatia experienced a rapid shift towards GLP-1 RA-based antihyperglycemic pharmacotherapy, with GLP-1-based therapies exerting a disproportionate budget impact. For payers, these surveillance data support budget forecasting and negotiation of pricing and reimbursement conditions; clinicians can use them to benchmark and optimize evidence-aligned prescribing; and policymakers can apply them to monitor the diffusion and fiscal impact of high-cost therapies. Routine national ATC/DDD analysis, complemented by HZZO claims and primary-care datasets, is essential for guiding future pricing, reimbursement and formulary decisions.

## 1. Introduction

Glucagon-like peptide-1 (GLP-1) receptor agonists and the dual GLP-1/GIP agonist have reshaped the therapeutic management of type 2 diabetes by influencing international treatment guidelines and prescribing patterns, rather than influencing patient-level outcomes directly within the scope of this study. GLP-1 receptor agonists and, more recently, the dual GLP-1/GIP agonist have demonstrated robust glucose-lowering efficacy and cardiovascular benefits, leading to rapid global uptake [1,2]. Parallel growth in sodium–glucose cotransporter 2 (SGLT-2) inhibitors has further diversified the therapeutic options [3,4]. Although these innovations improve clinical outcomes, their high acquisition costs and expanding indications have created a new axis of pharmaceutical expenditure for antihyperglycemic care [5].

Systematic pharmacoepidemiologic evidence from smaller European Union health systems with constrained pharmaceutical budgets remains limited. National drug-utilization studies are essential to understand how innovation translates into population-level prescribing and to anticipate the budgetary implications for public health insurers. Croatia provides an informative setting: it maintains universal health coverage and centralized drug procurement, while the Croatian Agency for Medicinal Products and Medical Devices (HALMED) publish annual national consumption data based on the World Health Organization’s Anatomical Therapeutic Chemical and Defined Daily Dose (ATC/DDD) methodology. These reports enable a reproducible measurement of drug utilization in DDD/1000 inhabitants/day and expenditure in euros, supporting surveillance of therapeutic trends and stewardship of health system resources [6,7].

For policymakers, HALMED’s national ATC/DDD data are directly relevant to health system stewardship because they provide the only complete, routinely collected source of standardized information on the diffusion, intensity and budget impact of antihyperglycemic therapies across all care settings. In a system with centralized procurement and a single dominant public payer, these data enable monitoring of how rapidly high-cost agents enter clinical practice, whether prescribing patterns align with guideline recommendations, and how therapeutic shifts translate into fiscal pressure on the pharmaceutical budget. Such surveillance is essential for anticipating reimbursement needs, informing price negotiations and ensuring that resource allocation keeps pace with evolving clinical evidence and population demand.

Comparative European data demonstrate marked heterogeneity in the uptake of novel antihyperglycemic therapies. Analyses of 11 EU countries show that high-income systems such as Austria, Germany, Sweden and the United Kingdom achieved substantially higher volume market shares and annual therapy costs for GLP-1 receptor agonists and other new antidiabetic medicines than Central and Eastern European (CEE) countries, including Croatia, over the first decade after market entry [8]. More recent multinational and global drug-utilization studies confirm that the use of GLP-1 RAs and SGLT-2 inhibitors remains concentrated in high-income health systems, whereas most CEE countries report lower per capita consumption, despite rising trends [5,9,10]. These differences in uptake and budget impact underscore the value of country-level analyses, such as the present study, for positioning Croatia within broader European diffusion patterns.

Previous HALMED bulletins have documented overall growth in antihyperglycemic drug consumption but without analytical interpretation by therapeutic subclass [6]. No publication has quantified the diffusion of GLP-1 receptor agonists or the dual GLP-1/GIP agonist in Croatia or assessed their contribution to total antihyperglycemic drug spending. Such evidence is increasingly relevant for health system sustainability and for comparative assessments across European Union member states.

In Croatia, the dual GLP-1/GIP agonist (ATC A10BX16) was not marketed before 2024; therefore, utilization and expenditure trends for 2017–2023 reflect GLP-1 receptor agonists only, with dual agonist entry captured only in the final study year.

To our knowledge, this is the first peer-reviewed analysis to quantify the uptake and budget impact of GLP-1 receptor agonists and the dual GLP-1/GIP agonist in Croatia. It also provides some of the earliest national-level data on the real-world entry of the dual GLP-1/GIP agonist in Central and Eastern Europe.

Within the broader antihyperglycemic armamentarium, GLP-1 receptor agonists (A10BJ) and the dual GLP-1/GIP agonist (A10BX16) are of particular interest for health system stewardship. They combine high acquisition costs with rapidly expanding indications across type 2 diabetes, obesity and metabolic dysfunction-associated steatotic liver disease, and they have become central to contemporary cardiovascular and renal risk-reduction strategies. These agents also account for a disproportionate share of recent growth in diabetes-drug expenditure in Croatia, whereas older incretin-related agents such as DPP-4 inhibitors (A10BH) are largely generic, associated with more modest incremental clinical benefit and did not materially drive the observed expenditure increase. For these reasons, we focused our detailed analyses on GLP-1 receptor agonists and the dual GLP-1/GIP agonist, while retaining DPP-4 inhibitors within the residual “other oral A10B” category.

The present study therefore aimed to (i) describe national utilization and expenditure trends for antihyperglycemic drugs (ATC A10) from 2017 through to 2024; (ii) quantify growth in GLP-1 receptor agonists (A10BJ) and the dual GLP-1/GIP agonist (A10BX16), relative to insulins and their analogs (A10A) and SGLT-2 inhibitors (A10BK); and (iii) estimate their cumulative budget impact within total antihyperglycemic drug expenditure.

## 2. Materials and Methods

### 2.1. Data Source and Setting

HALMED annual drug-consumption reports provide aggregate national data for all medicines that were in trade in Croatia in each calendar year. For each product, utilization is reported as defined daily doses per 1000 inhabitants per day (DDD/1000/day) and expenditure as the total value in EUR at wholesale prices. In this study, we used the total national figures, which therefore reflect medicines dispensed in outpatient and hospital sectors. Expenditure figures in HALMED reports represent wholesale pharmacy purchase prices (list prices), rather than retail payer prices or reimbursed amounts. These values do not capture confidential discounts, rebates or final expenditures borne by the Croatian Health Insurance Fund (HZZO).

### 2.2. Classification and Measurement

Drugs were identified according to the World Health Organization Anatomical Therapeutic Chemical (ATC) system and expressed in defined daily doses (DDD) per 1000 inhabitants per day (DDD/1000/day). The analysis covered total antihyperglycemic agents (A10—drugs used in diabetes) and the following therapeutic subgroups:

A10A—Insulins and analogs.
A10B—Oral blood-glucose-lowering drugs.
A10BK—Sodium–glucose cotransporter 2 (SGLT-2) inhibitors.
A10BJ—GLP-1 receptor agonists.
A10BX16—dual GLP-1/GIP agonist (single agent).

The dual GLP-1/GIP agonist (A10BX16) was not marketed in Croatia before 2024; HALMED reports show no utilization of this class in 2017–2023.

For each calendar year, the total A10 group was used to compute relative shares.

For once-weekly GLP-1 receptor agonists and the newly introduced dual GLP-1/GIP agonist, the WHO-defined DDD may not correspond to real-world therapeutic doses, which vary by indication, titration schedule and formulation. Consequently, DDD-based utilization reflects standardized population-level consumption, rather than the number of treated patients or true clinical dosing. This limitation is inherent to the ATC/DDD methodology and should be considered when interpreting utilization trends.

The WHO Collaborating Centre revised the DDD for liraglutide (A10BJ02) from 1.2 mg P to 1.5 mg P in 2020 [11]. To ensure comparability over time, utilization for A10BJ02 was recalculated for all pre-2020 years, using the updated DDD. Because A10BJ02 contributes to multiple aggregated categories, all affected aggregates (A10BJ, A10B and total A10) were recalculated using the harmonized DDD set.

### 2.3. Variables

Two primary variables were analyzed:

Utilization—DDD/1000 inhabitants/day, reflecting population-standardized consumption.
Expenditure—annual cost in euros (EUR) at current prices.

### 2.4. Derived Indicators

Old vs. new agents:

“New agents” = GLP-1 receptor agonists (A10BJ) + dual GLP-1/GIP agonist (A10BX16).

“Old agents” = total A10 minus new agents.

Budget share (%): (new agents’ expenditure/total A10 expenditure) × 100.

Compound annual growth rate (CAGR): (*V_n_*/*V*_0_)^1/*n*^ − 1, calculated from the first non-zero year for each class.

The binary categorization of “old” versus “new” antihyperglycemic agents was introduced to distinguish recently marketed, high-cost incretin-based injectables—GLP-1 receptor agonists (A10BJ) and the dual GLP-1/GIP agonist (A10BX16)—from the remainder of A10 therapies, which consist largely of long-established, lower-cost oral or insulin formulations. These newer incretin therapies differ fundamentally from older A10 agents in terms of acquisition cost, route of administration, therapeutic positioning and the speed and magnitude of their budget impact. Grouping A10BJ and A10BX16 together therefore provides a coherent analytical category, capturing the principal contemporary drivers of expenditure growth, while the “old agents” category reflects therapies with stable utilization patterns and limited incremental budget effect.

### 2.5. Data Processing and Analysis

All data were manually verified against the original HALMED tables and harmonized into a single wide-format dataset (data-extracted-glp_v1.xlsx). Analyses were performed in R (version 4.5.2), using the tidyverse and scales packages. Figures display trends for utilization and expenditure by ATC subclass, total A10 versus A10 excluding new agents and class composition by year. All utilization series reflect the updated post-2020 DDD definitions for consistency across the full 2017–2024 period.

Given the aggregate structure of the HALMED dataset, analyses were intentionally limited to descriptive statistics (annual levels, relative shares and compound annual growth rates); no patient-level modeling or hypothesis testing was undertaken.

A sensitivity analysis deflating expenditures to constant 2024 euros using Croatia’s Harmonized Index of Consumer Prices (HICP, Eurostat dataset) is shown in Supplement Table S1.

Prior to 2023, HALMED reports list expenditure in Croatian kuna (HRK). We converted HRK to EUR using the official irrevocable conversion rate (1 EUR = 7.53450 HRK), then deflated nominal EUR to constant 2024 euros using Croatia’s Harmonized Index of Consumer Prices (HICP; base 2024 = 100). The deflation formula was as follows: Real €_2024,t_ = Nominal €_t_ × (100/HICP_t_). The sensitivity results are shown in Table S1. We report both nominal and real amounts; figures explicitly label the price basis. Using a single, economy-wide deflation index (HICP) over an eight-year period introduces limitations. The HICP reflects general consumer price inflation and may not fully capture pharmaceutical-specific price dynamics, including confidential rebates, reference-pricing adjustments, or tender-driven price changes. As a result, real-term expenditure estimates should be interpreted as approximations of underlying price trends, rather than precise measures of net drug-cost evolution.

Reporting follows the STROBE statement for observational studies. No human subjects or identifiable information were involved; therefore, an ethics review was not required.

Use of GenAI in writing: Generative AI tools (ChatGPT 5.1) were used to assist with language editing and clarity improvements. All content, analysis, and interpretations were developed by the authors, who reviewed and approved the final text.

## 3. Results

### 3.1. Overall Utilization and Expenditure Trends

**Figure 1:**
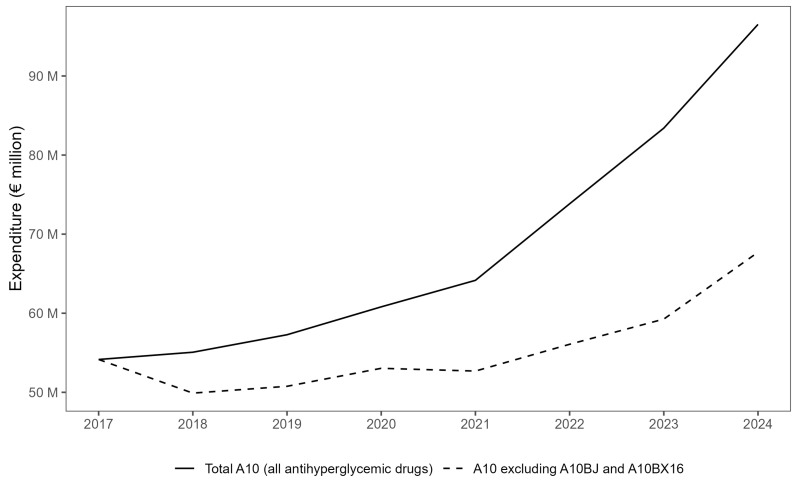
Annual antihyperglycemic drug expenditure (ATC A10) in Croatia, 2017–2024, compared with A10 expenditure, excluding GLP-1 receptor agonists (A10BJ) and the dual GLP-1/GIP agonist (A10BX16). Values expressed in euros (EUR million).

Between 2017 and 2024, total utilization of antihyperglycemic drugs (ATC A10) in Croatia increased from 66.9 to 96.8 DDD/1000 inhabitants/day, representing a relative rise of 44.7%. Over the same period, total expenditure grew from EUR 54 million to EUR 96 million (Figure 1), corresponding to an average annual increase (CAGR) of 8.6%. Although the overall trajectory was upward, a small fluctuation is visible in 2018, reflecting minor year-to-year variation in list prices and class composition; however, this deviation does not alter the clear multi-year trend of rising national expenditure, which accelerated markedly after 2021.

Over the same period, estimates from the International Diabetes Federation indicate a continued increase in the number of adults living with diabetes in Croatia; thus, part of the observed 44.7% rise in DDD/1000 inhabitants/day is likely driven by a growing patient population. However, as later discussed, the disproportionate expansion of GLP-1 receptor agonists and SGLT-2 inhibitors and their rising budget share suggest that changes in prescribing intensity and class mix, rather than prevalence growth alone, also contributed to higher utilization.

The steeper year-on-year increases observed after 2021 are consistent with the cumulative impact of international guideline updates and indication expansions that prioritized GLP-1 receptor agonists and SGLT-2 inhibitors in patients with high cardiovascular and renal risk, as well as increasing availability and familiarity with once-weekly GLP-1 formulations in routine practice. In the Croatian context, these developments likely translated into broader prescribing of novel agents, amplifying their contribution to total A10 expenditure.

### 3.2. Class-Specific Trends

**Figure 2:**
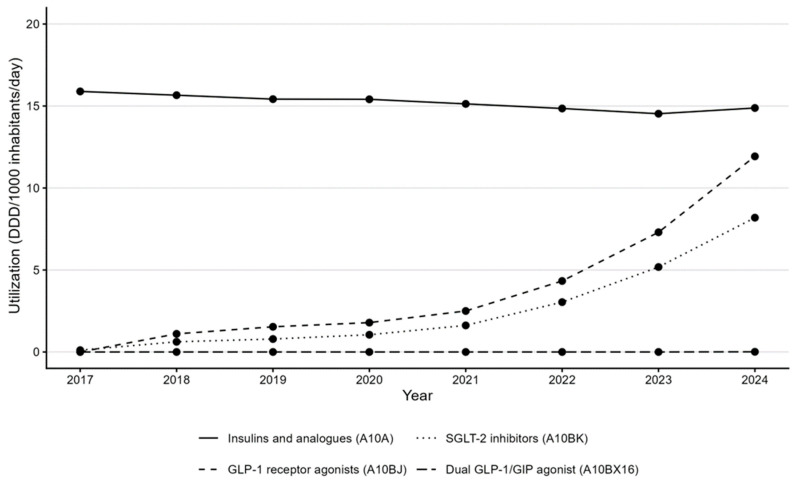
Utilization of antihyperglycemic drugs in Croatia, 2017–2024, by ATC subclass: insulins and analogs (A10A), GLP-1 receptor agonists (A10BJ), SGLT-2 inhibitors (A10BK) and dual GLP-1/GIP agonist (A10BX16). Utilization is expressed as DDD per 1000 inhabitants per day (DDD/1000/day).

**Figure 3:**
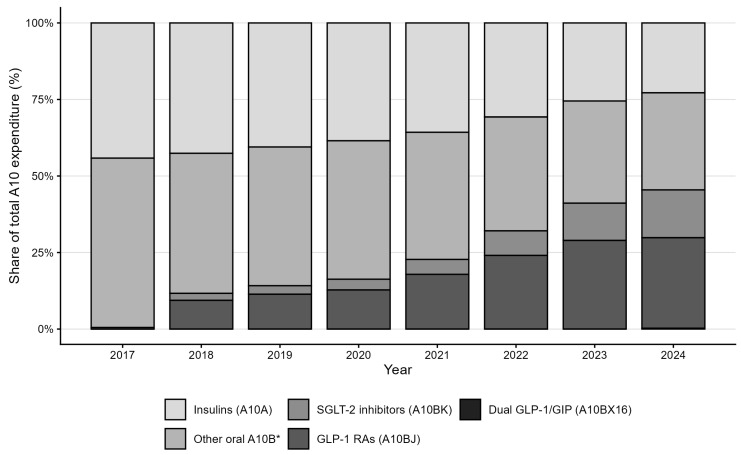
Composition of national antihyperglycemic drug expenditure (ATC A10) in Croatia, 2017–2024, by mutually exclusive therapeutic subclasses: insulins (A10A), other oral A10B* agents, SGLT-2 inhibitors (A10BK), GLP-1 receptor agonists (A10BJ) and dual GLP-1/GIP agonist (A10BX16). Values represent each subclass’ percentage share of total A10 expenditure. * Other oral A10B agents exclude A10BK, A10BJ and A10BX16.

Insulins and analogs (A10A) remained a major therapeutic category but showed a minor change in utilization (15.9 → 14.9 DDD/1000/day) (Figure 2) and stable expenditure (between EUR 21.3 million and EUR 23.9 million in the period between 2017 and 2024) (Figure 3).

The A10BX16 subgroup first appeared in Croatian national data in 2024, with very low utilization (<0.05 DDD/1000/day) and EUR 0.3 million in expenditure, reflecting its late market entry and limited reimbursement during the study window.

SGLT-2 inhibitors (A10BK) expanded rapidly from 0.1 DDD/1000/day in 2017 to 8.2 DDD/1000/day in 2024, with expenditure increasing from EUR 0.3 million to EUR 15.1 million, equivalent to a CAGR of 76.5% from their first year on the market.

GLP-1 receptor agonists (A10BJ) increased from 1.1 DDD/1000/day in 2018 (year of introduction) to 11.9 DDD/1000/day in 2024, and from EUR 5.2 million to EUR 28.6 million in expenditure (CAGR of 33.0%).

The faster growth in the GLP-1 receptor agonist expenditure compared with SGLT-2 inhibitors, despite lower DDD volumes, reflects both higher per-DDD acquisition costs for injectable GLP-1-based therapies and the early uptake of once-weekly, higher-dose formulations. Consequently, each additional unit of GLP-1 utilization carries a greater budget impact than an additional DDD of SGLT-2 inhibitors, producing steeper expenditure trajectories, even when volume growth is more modest.

A10BX16 appeared in 2024, contributing EUR 0.3 million and 0.01 DDD/1000/day in its first year of availability.

### 3.3. Shift in Budget Composition

**Figure 4:**
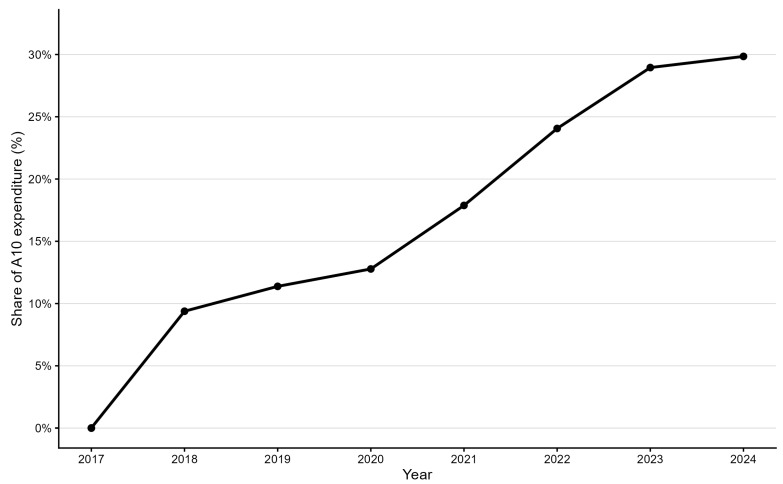
Share of GLP-1 receptor agonists (A10BJ) and dual GLP-1/GIP agonist (A10BX16) in total national antihyperglycemic drug expenditure (ATC A10), Croatia 2017–2024. Values represent percentage of total A10 expenditure.

The relative share of GLP-1 RA and the dual GLP-1/GIP agonist within total antihyperglycemic drug expenditure increased from zero in 2017 to 29.8% in 2024 (Figure 3). Insulins represented about 23% of total A10 expenditure in 2024, while SGLT-2 inhibitors accounted for 15.6%. The residual oral and combination therapies collectively declined from more than half of the total in 2017 to 31.7% in 2024. The year-by-year evolution of the expenditure share of GLP-1 RA and dual GLP-1/GIP agonist therapies is shown in Figure 4.

Despite broadly similar growth in standardized utilization, SGLT-2 inhibitors account for a substantially smaller share of total A10 expenditure than GLP-1 receptor agonists and the dual GLP-1/GIP agonist. This divergence reflects pronounced differences in acquisition cost: per-DDD prices for injectable GLP-1-based therapies are considerably higher than for oral SGLT-2 inhibitors. As a result, comparable increases in DDD/1000 inhabitants/day translate into a much larger budget impact for GLP-1-based agents than for SGLT-2 inhibitors.

### 3.4. Utilization Patterns

The GLP-1 RA and dual GLP-1/GIP agonist therapies’ volume share reached 12.3% of the total A10 DDD in 2024, whereas insulin use remained stable and other oral agents declined slightly. Figure 2 demonstrates consistent upward trajectories for A10BJ and A10BK classes, with a discernible acceleration after 2021.

The parallel increase in both DDDs and expenditure indicates that market expansion, rather than price inflation, was the primary driver of budget growth.

### 3.5. Summary Indicators

Table 1 presents annual expenditure data for major A10 subclasses, and Table 2 presents annual utilization. Table 3 summarizes compound annual growth rates and changes in budget share between 2017 and 2024. Total antihyperglycemic drug expenditure grew 8.6% per year, GLP-1 receptor agonist expenditure grew 33% per year from 2018 and SGLT-2 inhibitor expenditure grew 76% per year from 2017.

Across 2017–2024, GLP-1 receptor agonists and the dual GLP-1/GIP agonist increased their expenditure share from 0% to 29.8%, confirming their emergence as the dominant growth driver within Croatia’s antihyperglycemic pharmacotherapy market.

Of the EUR 42.3 million increase in total A10 expenditure between 2017 and 2024, A10BJ plus A10BX16 accounted for EUR 28.9 million (68%), and SGLT-2 inhibitors for EUR 14.8 million (35%). The sum exceeds 100% because insulin expenditure declined slightly over the period.

After deflation to constant 2024 euros, total A10 expenditure increased from EUR 71 million (2017) to EUR 96.5 million (2024), yielding a real CAGR of 4.5% (Table S1).

## 4. Discussion

This national pharmacoepidemiologic analysis shows a marked transformation in the utilization and expenditure of antihyperglycemic drugs in Croatia from 2017 through to 2024. Over these eight years, total antihyperglycemic drug spending increased by nearly 78%, with most of the expansion being attributable to GLP-1 receptor agonists and dual GLP-1/GIP agonist. These classes accounted for roughly one-third of total A10 expenditure in 2024, despite representing a modest share of DDD consumption. The findings reflect a structural shift in Croatia’s antihyperglycemic pharmacotherapy market, paralleling international patterns of rapid uptake of high-cost, high-impact therapies. In this context, a “structural shift” refers to a measurable re-composition of the national A10 market from long-established insulins and low-cost oral agents towards newer, high-cost GLP-1 RA, the dual GLP-1/GIP agonist and SGLT-2 therapies. Between 2017 and 2024, the combined expenditure share of GLP-1 receptor agonists and the dual GLP-1/GIP agonist increased from 0% to 29.8%, while SGLT-2 inhibitors rose to 15.6% of total A10 expenditure; over the same period, the residual oral A10B group declined from more than half of total spending to 31.7%, and insulin expenditure remained broadly stable at around one quarter of the total (Table 1, Figure 3). These changes indicate that recent growth in Croatia’s diabetes-drug budget is driven predominantly by the rapid diffusion of high-cost incretin-based and SGLT-2 agents, rather than by proportional increases in older oral therapies or insulins. Our decision to highlight GLP-1 receptor agonists and the dual GLP-1/GIP agonist reflects their role as the principal recent drivers of A10 expenditure and policy concern, in contrast to DPP-4 inhibitors, which remain embedded in the background of older oral A10B therapies with comparatively limited marginal budget impact.

The A10BX16 subclass first appears in the Croatian national utilization data in 2024, corresponding to the initial market entry of the dual GLP-1/GIP agonist. Although the agent had received European regulatory approval earlier, it only became available in Croatia late in the study window and without reimbursement, requiring out-of-pocket payment. Consequently, the recorded utilization in 2024 was minimal (<0.05 DDD/1000/day) and its contribution to overall expenditure was negligible. These patterns are consistent with the expected early-adoption dynamics of a high-cost therapy introduced outside the public insurance system.

Croatia’s rapid uptake of GLP-1 receptor agonists and the dual GLP-1/GIP agonist mirrors Western European trends, despite its smaller population, budget constraints and later market entry, with GLP-1 RAs and SGLT-2 inhibitors expanding sharply in both volume and expenditure. This growth is driven by broadening clinical indications, following cardiovascular and renal-outcome evidence [12,13,14,15]. The observed pattern is consistent with predominantly additive growth in antihyperglycemic drug spending, rather than wholesale substitution of older agents. Total A10 expenditure increased from EUR 54.2 million to EUR 96.5 million between 2017 and 2024, while insulin utilization remained broadly stable, the residual oral A10B group retained a substantial volume share, and GLP-1 receptor agonists and SGLT-2 inhibitors expanded rapidly in both DDDs and budget share (Table 1 and Table 2, Figure 2 and Figure 3). These aggregate trends suggest that novel agents have largely been layered onto, rather than fully replacing existing therapies, although patient-level data would be required to distinguish add-on use from switching with certainty. If recent trajectories persist, the budget share of GLP-1 receptor agonists and dual GLP-1/GIP agonist is likely to increase further, potentially approaching one half of total A10 spending over the medium term; however, this depends on uptake, indication expansion and pricing dynamics. Recent national surveys of Croatian general practitioners confirm that GLP-1 receptor agonists and the dual GLP-1/GIP agonist remain underutilized in primary care: GLP-1 receptor agonists are prescribed to only about 14% of patients and SGLT-2 inhibitors to 21%, indicating that many eligible individuals still do not receive these cardioprotective treatments [16,17].

These patterns underscore the need for real-time monitoring to guide pricing and reimbursement policy, especially considering these drugs are emerging as a promising option for the obesity and metabolic dysfunction-associated steatotic liver disease (MASLD) with fibrosis, which might substantially consume the healthcare budget [18,19,20,21]. Building on this expanding therapeutic scope, growing evidence demonstrates that dual GIP/GLP-1 receptor agonists achieve substantial and sustained weight loss in non-diabetic obese individuals, suggesting that clinical demand for this class will continue to rise and further intensify budgetary pressures [22]. While the study benefits from complete national data and standardized metrics, its aggregate design limits inference because HALMED data lack patient-level detail, differentiate neither care settings nor true reimbursed costs and rely on DDD units that may not reflect real-world dosing. As a result, the analytical depth and clinical interpretability of our findings are necessarily restricted to system-level patterns in drug utilization and expenditure, rather than individual treatment effects or outcomes.

The acceleration in the use of GLP-1 receptor agonists and the dual GLP-1/GIP agonist use observed after 2021 likely reflects the impact of international guideline updates and expanded indications. In particular, the 2018 ADA–EASD consensus report and subsequent updates recommended GLP-1 receptor agonists and SGLT-2 inhibitors for patients with established cardiovascular disease or high-risk profiles, regardless of HbA1c levels. The European Medicines Agency (EMA) approvals for new cardiovascular and renal indications between 2019 and 2022 also expanded their eligibility. These developments likely influenced national prescribing behavior, even in the absence of formal changes to Croatian reimbursement criteria. Reimbursement of once-weekly GLP-1 RAs and broader formulary access may also have facilitated uptake in later years. Despite these gains, real-world implementation in primary care remains incomplete.

Although international consensus statements and cardiovascular-outcome evidence clearly shaped therapeutic expectations in Croatia, national professional guidance also played a role. The Croatian Society for Diabetes and Metabolic Disorders of the Croatian Medical Association has, since 2016, regularly issued expert recommendations on the use of oral and injectable antihyperglycemic agents, which were disseminated at national diabetes congresses and communicated to the Croatian Health Insurance Fund (HZZO) for consideration in reimbursement decisions. These recommendations have guided clinical decision-making in practice, but they were not published as publicly available formal documents and therefore cannot be cited directly. A new consolidated national guideline is currently in final preparation and is expected to be published in early 2026. Consequently, the observed utilization trends during 2017–2024 reflect the combined influence of evolving international evidence and locally disseminated expert society recommendations, even though no formally updated national guideline was issued during the study window.

Comparable trends have been documented in neighboring and regional health systems, with Western and Northern European countries generally reporting an earlier and steeper uptake of GLP-1 RAs and SGLT-2 inhibitors than CEE countries [5,8,10]. Within CEE, Hungary and others show dynamic growth of novel antidiabetic drugs but persistently substantial use of older oral agents, reflecting the structural and budgetary constraints [5,10]. Against this backdrop, Croatia’s 2017–2024 trajectory—where GLP-1 receptor agonists and the dual GLP-1/GIP agonist already account for nearly one-third of A10 expenditure—suggests convergence towards higher-uptake EU systems, despite a later market entry. Croatia’s trajectory therefore aligns closely with regional patterns despite the later market entry and a more constrained pharmaceutical budget. This convergence suggests that evidence from cardiovascular and renal outcome trials exerted similar prescribing pressure across Central Europe, overriding structural differences in the procurement and reimbursement systems.

In a universal coverage system with a single dominant public payer, rapid uptake of high-cost therapies has direct implications for pharmaceutical budget sustainability. The shift toward GLP-1 RAs and SGLT-2 inhibitors—combined with expanding cardiovascular-, renal- and obesity-related indications—creates upward expenditure pressure that may require future adjustments in pricing negotiations, reimbursement criteria or therapeutic guidelines. The Croatian system’s constrained fiscal envelope means that continued diffusion of GLP-1 receptor agonists and the dual GLP-1/GIP agonist must be balanced against competing health system priorities.

This study provides timely, actionable evidence for national payers and policymakers navigating the budgetary implications of high-cost therapies with expanding indications. As such, it offers a case study for similar smaller European health systems with limited fiscal space for pharmaceuticals that are managing therapeutic innovation under resource constraints.

Several limitations must be acknowledged. HALMED reports provide aggregate national data that do not distinguish patient characteristics, diabetes duration, clinical indications, or treatment lines. Expenditure figures reflect the published prices and may not capture confidential rebates or negotiated discounts. DDD values do not necessarily correspond to real-world dosing regimens for agents such as GLP-1 receptor agonists, potentially over- or underestimating true consumption. Hospital and outpatient channels are aggregated, preventing sector-specific analysis. Finally, this study does not adjust for changes in diabetes prevalence or therapeutic guidelines across the observation period. Despite these constraints, the complete national coverage and the standardized ATC/DDD methodology ensure strong internal consistency and comparability across years.

Temporal changes in pharmaceutical policy may have influenced the observed trends but could not be disentangled in this analysis. HALMED reports capture the final aggregate effect of prescribing behavior, reimbursement criteria, reference pricing and tender outcomes on national consumption and list-price expenditure; however, they do not include explicit information on when such policy changes occurred or how strongly they affected specific subclasses. As a result, we cannot determine whether annual fluctuations or inflection points reflect shifts in clinical demand, alterations in reimbursement or prescribing restrictions, or tender-driven price changes. The trends should therefore be interpreted as descriptive system-level patterns, rather than causal reflections of specific policy or pricing interventions.

To avoid denominator-driven bias arising from mid-period DDD revisions, we harmonized the dataset by recalculating A10BJ02 and all affected aggregates (A10BJ, A10B and total A10), using the updated DDD.

We did not adjust utilization rates for temporal changes in diabetes prevalence. Data from the International Diabetes Federation (IDF) indicate that the absolute number of adults with diabetes in Croatia increased from roughly 0.22 million in 2011 to approximately 0.41 million in 2024 [23]. This suggests that part of the observed increase in DDD/1000 inhabitants/day reflects a growing patient population. However, even accounting for this rise in case numbers, the relative expansion of GLP-1 receptor agonists and SGLT-2 inhibitors and their disproportionate contribution to total A10 expenditure indicates a genuine intensification and shift in pharmacotherapy, rather than a purely prevalence-driven effect.

This descriptive national analysis also highlights several research priorities. First, linking the HALMED ATC/DDD data with the Croatian Health Insurance Fund’s (HZZO) claims and primary-care electronic health records would enable patient-level analyses of indication, treatment lines and appropriateness of GLP-1 and SGLT-2 use, including equity in access across age, sex and region. Second, longitudinal cohort studies using such linked datasets are needed to assess whether the increased utilization of novel antihyperglycemic agents translates into measurable improvements in cardiovascular, renal and liver outcomes at the population level. Third, formal budget-impact and cost-effectiveness models should quantify the medium-term fiscal implications of expanding GLP-1-based and SGLT-2 indications, including obesity and MASLD, under alternative reimbursement and pricing scenarios. Finally, cross-country comparative studies employing harmonized ATC/DDD methodologies could position Croatia’s trajectory within a broader European context and identify structural features of health systems that facilitate or constrain a clinically appropriate, fiscally sustainable uptake of high-cost antihyperglycemic drugs.

## 5. Conclusions

Croatia’s antihyperglycemic drug landscape underwent a marked transformation between 2017 and 2024, characterized by the rapid expansion of GLP-1 receptor agonists and the dual GLP-1/GIP agonist and sustained growth of SGLT-2 inhibitors. These therapies now represent the principal drivers of expenditure growth and have significantly reshaped the composition of national diabetes pharmacotherapy. Inflation-adjusted results confirm that market expansion—not price inflation—was the dominant contributor to rising costs.

Given their escalating budget impact, Croatia requires structured, class-specific monitoring of antihyperglycemic drugs. At a minimum, this should include (i) annual national ATC/DDD analyses from HALMED that report utilization and expenditure separately for insulins, older oral agents, SGLT-2 inhibitors and GLP-1-based therapies; (ii) internal HZZO dashboards that track quarterly expenditure for high-cost classes against budget projections; and (iii) periodic linkage of HALMED aggregates with reimbursement and primary-care data to identify shifts in prescribing patterns and potential over- or under-use in high-risk populations. Such targeted monitoring would enable Croatian decision-makers to detect rapid changes in uptake, anticipate budget pressures and align pricing and reimbursement decisions with evolving clinical evidence.

Routine analysis of HALMED ATC/DDD data is therefore a necessary foundation for pharmaceutical stewardship, but it is not sufficient on its own for fully informed policy decisions. For reimbursement design, assessment of appropriate use and evaluation of clinical outcomes, HALMED aggregates need to be complemented by HZZO claims data and, where feasible, primary-care electronic health records. Only the combination of these sources can show not just how much of each class is used and at what cost, but which patients receive these therapies, under which indications and with what downstream consequences for health outcomes and resource use.

In practical terms, these findings support several policy responses for Croatia and comparable health systems. First, HZZO could periodically review reimbursement criteria for GLP-1-based and SGLT-2 therapies to ensure that coverage prioritizes patients at the highest cardiovascular-, renal- or obesity-related risk, thereby maximizing clinical value per euro spent. Second, national budget impact analyses based on HALMED and HZZO data should be used in price negotiations and, where appropriate, in designing risk-sharing or outcome-based agreements for high-cost incretin-based agents. Third, formulary committees and professional societies should use these utilization patterns to align local prescribing recommendations with international guidelines, while discouraging low-value use in lower-risk populations. Finally, tracking regional and provider-level variation in the uptake of GLP-1 and SGLT-2 therapies could help identify unwarranted underuse or overuse and inform targeted educational or regulatory interventions.

## Figures and Tables

**Table 1:** Annual expenditure for A10 subclasses, Croatia 2017–2024.

	A10A (Insulins)	Other Oral A10B ^1^	SGLT-2 Inhibitors (A10BK)	GLP-1 RAs (A10BJ)	Dual GLP-1/GIP Agonist (A10BX16)	Total A10
2017	23.9	30.0	0.3	0.0	0.0	54.2
2018	23.4	25.2	1.3	5.2	0.0	55.1
2019	23.2	26.0	1.6	6.5	0.0	57.3
2020	23.4	27.5	2.1	7.8	0.0	60.8
2021	22.9	26.6	3.1	11.5	0.0	64.2
2022	22.7	27.5	5.9	17.8	0.0	73.8
2023	21.3	27.8	10.2	24.1	0.0	83.4
2024	22.0	30.6	15.1	28.6	0.3	96.5

^1^ Other oral A10B excludes A10BK, A10BJ and A10BX16. Notes: Expenditure = nominal euros (EUR million); constant-2024-euro sensitivity in Table S1. Columns may not sum exactly, due to rounding. Source: HALMED annual drug-consumption reports; authors’ calculations. A10BX16 (dual GLP-1/GIP agonist) was not marketed before 2024; no utilization is recorded for 2017–2023.

**Table 2:** Annual utilization for A10 subclasses, Croatia 2017–2024.

	A10A (Insulins)	Other Oral A10B ^1^	SGLT-2 Inhibitors (A10BK)	GLP-1 RAs (A10BJ)	Dual GLP-1/GIP Agonist (A10BX16)	Total A10
2017	15.9	50.9	0.1	0.0	0.0	66.9
2018	15.7	51.6	0.6	1.1	0.0	69.0
2019	15.4	59.8	0.8	1.5	0.0	77.6
2020	15.4	61.3	1.0	1.8	0.0	79.5
2021	15.1	56.2	1.6	2.5	0.0	75.5
2022	14.8	58.1	3.0	4.3	0.0	80.3
2023	14.5	59.3	5.2	7.3	0.0	86.3
2024	14.9	61.8	8.2	11.9	0.0 ^2^	96.8

^1^ Other oral A10B excludes A10BK, A10BJ and A10BX16. ^2^ In 2024, Dual GLP-1/GIP utilization < 0.05 DDD/1000/day; displayed as 0.0, due to rounding. A10BX16 was not marketed before 2024; no utilization is recorded for 2017–2023. Note: Utilization = DDD per 1000 inhabitants per day (DDD/1000/day). Columns may not sum exactly, due to rounding.

**Table 3:** Compound annual growth rates (CAGR) by class and budget share change, 2017–2024.

Antihyperglycemic Drug Expenditure Trends, Croatia 2017–2024	
Total A10 expenditure CAGR	8.6%
GLP-1 expenditure CAGR	33.0%
GLP-1 growth period	2018–2024
SGLT-2 expenditure CAGR	76.5%
SGLT-2 growth period	2017–2024
Share of new drugs (2017)	0.0%
Share of new drugs (2024)	29.8%

CAGR = Compound annual growth rate; new drugs = GLP-1 receptor agonists (A10BJ) + dual GLP-1/GIP agonist (A10BX16).

## Data Availability

The original data and analysis script presented in the study are openly available on the Open Science Framework (OSF) at https://doi.org/10.17605/OSF.IO/Y2JS5.

## Supplementary Materials

The following supporting information can be downloaded at: https://www.mdpi.com/article/10.3390/medicina61122210/s1. Table S1: Sensitivity analysis of total A10 expenditure in nominal and constant 2024 euros.

## Abbreviations

The following abbreviations are used in this manuscript:

HALMED	Croatian Agency for Medicinal Products and Medical Devices
RA	Receptor agonists (e.g., GLP-1 RA)
GLP-1	Glucagon-like peptide-1
GIP	Glucose-dependent insulinotropic polypeptide
DDD	Defined daily dose
MASLD	Metabolic dysfunction-associated steatotic liver disease
SGLT-2	Sodium–glucose cotransporter 2
HZZO	Croatian Health Insurance Fund
ATC	Anatomical Therapeutic Chemical
CEE	Central and Eastern Europe
IDF	International Diabetes Federation
HICP	Harmonized Index of Consumer Prices
