# Supplementary material for: National Utilization and Expenditure Trends of GLP-1 Receptor Agonists and Dual GLP-1/GIP Agonist in Croatia, 2017–2024
Source: Medicina (Kaunas). 2025 Dec 15;61(12):2210. doi: 10.3390/medicina61122210 (PMC12734843; doi:10.3390/medicina61122210)
Supplement: Supplementary file 1 [file medicina-61-02210-s001.zip › medicina-4025037-supplementary.pdf]

Table S1. Sensitivity analysis of total A10 expenditure in nominal and constant 2024 euros

Year	Nominal EUR	HICP Index (2024 = 100)	Real EUR 2024
2017	54 154 165	76	71 083 170
2018	55 067 849	77	71 144 173
2019	57 280 562	78	73 415 533
2020	60 807 226	78	77 935 599
2021	64 158 742	80	80 069 310
2022	73 837 867	89	83 241 857
2023	83 406 162	96	86 742 409
2024	96 520 769	100	96 520 769

Notes: Nominal values are expressed in euros. Real values are converted to constant 2024 euros using Croatia's Harmonised Index of Consumer Prices (HICP) from Eurostat, re-based to 2024 = 100. Formula: $Real_t = Nominal_t \times (100 / HICP_t)$. Pre-2023 HRK amounts were converted to EUR at the official fixed rate (1 EUR = 7.53450 HRK) prior to deflation. Due to rounding, HICP values for 2019–2020 appear identical. Source: HALMED annual drug-consumption reports; authors' calculations.
